# *TRIB1* and *TRPS1* Gene Polymorphisms Are Associated with the Incidence of Acute Coronary Syndrome and Plasma Lipid Concentrations

**DOI:** 10.3390/biology14060606

**Published:** 2025-05-26

**Authors:** Gilberto Vargas-Alarcón, Óscar Pérez-Méndez, Rosalinda Posadas-Sánchez, Héctor González-Pacheco, Teresa Juárez-Cedillo, Galileo Escobedo, Victoria López-Olmos, José Manuel Fragoso

**Affiliations:** 1 Departamento de Biología Molecular, Instituto Nacional de Cardiología Ignacio Chávez, Juan Badiano No. 1, Tlalpan, Mexico City 14080, Mexico; gvargas63@yahoo.com (G.V.-A.); opmendez@yahoo.com (Ó.P.-M.); vickyolmosunam@hotmail.com (V.L.-O.); 2 Departamento de Endocrinología, Instituto Nacional de Cardiología Ignacio Chávez, Mexico City 14080, Mexico; rossy_posadas_s@yahoo.it; 3 Unidad Coronaria, Instituto Nacional de Cardiología Ignacio Chávez, Mexico City 14080, Mexico; hectorglezp@hotmail.com; 4 Unidad de Investigación en Epidemiologia y Servicios de Salud-Área de Envejecimiento, Centro Médico Nacional Siglo XXI, Instituto Mexicano del Seguro Social, Mexico City 06720, Mexico; terezillo@yahoo.com.mx; 5 Dirección de Investigación y Laboratorio de Inmunometabolismo, Hospital General de Mexico, Dr. Eduardo Liceaga, Mexico City 06720, Mexico; gescobedog@msn.com

**Keywords:** susceptibility, acute coronary syndrome, transcriptional repressor GATA binding 1, tribbles pseudokinase 1

## Abstract

In recent years, data from genome-wide association studies (GWAS) have shown that the genes coding for transcriptional repressor GATA binding 1 (*TRPS1*) and tribbles pseudokinase 1 (*TRIB1*) play an important role in plasma lipid profiles and act as risk factors for coronary heart disease (CHD). The aim of this work was to explore whether single nucleotide polymorphisms (SNPs) in the *TRSP1* (rs231150 and rs2737229) and *TRIB1* (rs2980880 and rs2954029) genes are involved in acute coronary syndrome (ACS) and plasma lipid levels. We included 1262 patients diagnosed with ACS and 1051 controls. According to inheritance models, the minor alleles of the SNPs (rs2737229 *A*, rs2980880 *C*, and rs2954029 *T*) were associated with an increased incidence of ACS (*p* < 0.05). In a sub-analysis that included only the control subjects, the same minor allele frequency was associated with increased total cholesterol, HDL-cholesterol, and LDL-cholesterol levels and low triglyceride levels. In conclusion, rs2737229, rs2980880, and rs2954029 polymorphisms are associated with a risk of developing ACS and with elevated plasma lipid levels. Our results suggest that the *TRSP1* and *TRIB1* are implicated in the incidence of ACS through of increased of plasma lipid profile.

## Simple Summary

Coronary heart disease is the main cause of mortality in the world, so the early screening of patients at high risk is a priority. In this context, genome-wide association and case–control studies have revealed that the presence of polymorphic sites in the transcriptional repressor GATA binding 1 (rs231150 *A/T* and rs2737229 *A/C*) and tribbles pseudokinase 1 (rs2980880 *T/C* and rs2954029 *T/A*) genes are associated with a predisposition to CAD and with plasma lipid profile levels. Therefore, here, we investigated whether these polymorphisms are associated with CAD clinical events. Our findings demonstrated that rs2737229 *A/C*, rs2980880 *T/C*, and rs2954029 *T/A* polymorphisms were associated with the risk of acute coronary syndrome development and with changes in plasma lipid levels. Such associations suggest that these genes are involved in the risk of CAD, probably through an increase in plasma lipid levels.

## 1. Introduction

Acute coronary syndrome (ACS) is a consequence of background genetics, dyslipidemia, and the cardiovascular risk factors such as obesity, elevated blood pressure, and diabetes mellitus 2 (DM2), as well as sex, age, smoking, and body index mass (BMI), which play a fundamental role in the development of ACS [1,2,3]. Previous genome-wide association studies (GWAS) have shown that the *TRPS1* (transcriptional repressor GATA binding 1) and *TRIB1* (tribbles pseudokinase 1) genes located on chromosome 8q23-24 are associated with the predisposition to coronary artery disease (CAD) and with plasma lipid profile levels [4,5,6,7,8,9,10]. *TRSP1* encodes a transcription factor that binds specifically to GATA sequences, thereby repressing the expression of GATA-regulated genes [11,12]. Currently, the molecular mechanisms by which *TRSP1* influences plasma lipid levels are poorly understood. *TRSP1* attenuates forkhead box A1 (FOXA1) binding to the promoter region of *SCARB1,* with a consequent decrease in the scavenger receptor B1 (SR-BI) expression [13]. This decrease may affect the transport of cholesterol contained in LDL and HDL to the liver [14], inducing dyslipidemia. *TRIB1* encodes pseudoprotein kinase 1, which regulates the activation of MAP kinases as this protein binds to MAPK substrates but lacks enzymatic activity [15]. Therefore, TR1B1 has a role as a modulator of many biological processes and disease pathologies [15,16]. In addition, studies in mice demonstrated that the overexpression of Trib1 reduced cholesterol and triglyceride concentrations, whereas knockout (Trib1_LSKO) led to significant increases in plasma cholesterol and triglycerides and an increase in C/EBP-alpha protein levels in the liver [17]. On the other hand, TR1B1, upon interaction with Sin3A-associated protein 18 kDa (SAP18), induces the transcription of microsomal triglyceride transporter protein (MTTP) and apolipoprotein B (apoB), enhancing hepatic lipogenesis [18,19,20]. In this context, recent studies have suggested that some genetic variants present in the *TRPS1* (rs231150 *A/T* and rs2737229 *A/C*) and *TRIB1* (rs2980880 *T/C* and rs2954029 *T/A*) genes are associated with plasma lipid profile levels and cardiovascular diseases such as coronary artery disease (CAD), myocardial infarction, hypercholesterolemia, and metabolic diseases such as diabetic mellitus (DM2) [4,5,6,7,8,9,10]. Nevertheless, most studies have included principally Caucasian and Asian populations. Thus, it is necessary to analyze non-European populations in order to better understand the role of the genetics in the development of ACS.

In this context, according to the role of these genes in the catabolism of lipids, we propose that *TRSP1* and *TRIB1* gene polymorphisms are associated with the incidence of ACS and with plasma lipid concentrations. Therefore, the objective of this study was to investigate whether the rs231150 *A/T*, rs2737229 *A/C*, rs2980880 *T/C*, and rs2954029 *T/A* SNPs correlate with the risk of ACS and plasma lipid levels.

## 2. Materials and Methods

### 2.1. Characteristics of the Study Population

This study included 2313 Mexican mestizo individuals, including 1051 control individuals and 1262 patients diagnosed with ACS. Consecutive patients with unstable angina or myocardial infarction with or without elevated segment ST were recruited from July 2018 to November 2023 in the Coronary Care Unit. ACS was diagnosed based on the international guidelines [21,22], which included examining symptoms, electrocardiographic changes, and creatine phosphokinase-MB isoenzyme (CK-MB) and troponin I levels. Patients were excluded when heart failure, hepatic disease, thyroid dysfunction, cancer, or infectious or autoimmune diseases were detected. As comparison (control) group, we included 1051 individuals selected from the Genetics of Atherosclerosis Disease (GEA) Mexican cohort, recruited from June 2008 to January 2013 in our institute, as previously described [23]. Controls were non-symptomatic individuals, with no family history of coronary heart disease or heart failure, and with no coronary calcium score determined by computed tomography. The exclusion criteria were liver, renal, thyroid, or oncological preexisting diseases or diagnosed during the recruitment period [23]. To calculate the sample size in an unmatched case–control study, we used an alpha error of 0.05 with a power of 80% (OpenEpi available online, (http://www.openepi.com/SampleSize/SSCC.htm (accessed on 12 November 2023)). All individuals who agreed to participate signed a letter of informed consent. The study met the terms of the Declaration of Helsinki and was accepted by the Ethics and Research commissions of the Instituto Nacional de Cardiologia Ignacio Chavez, Mexico, under the number 23-1361.

### 2.2. Laboratory Analyses

Cholesterol, triglycerides, and glucose in the blood were measured using special kits from Randox Laboratories (Crumlin, UK) on a Beckman Coulter DU-730 analyzer. High-density lipoprotein-cholesterol (HDL-C) plasma levels were measured after the selective precipitation of the lipoproteins containing apo B with phosphotungstic acid-Mg^2+^ (HDL-cholesterol, SPINREACT—Girona, Spain). LDL-C levels were calculated using the Friedewald formula for blood samples with triglycerides levels under 400 mg/dL [24]. Dyslipidemia was defined as a cholesterol > 200 mg/dL or LDL-C > 130 mg/dL or HDL-C < 40 mg/dL or triglycerides > 150 mg/dL [25]. Patients were considered diabetic when their fasting glucose levels were > 125 mg/dL [26]. Technical personnel trained for this purpose determined blood pressure using the auscultatory method after 15 min of resting. The measurement was determined twice, and the reported blood pressure is the mean of both values. Elevated blood pressure was defined by systolic blood pressure ≥ 120 to 139 mmHg, diastolic blood pressure ≥ 70 to 89 mmHg, or oral antihypertensive therapy [27]. Anthropometric and demographic information was obtained from the medical record.

### 2.3. Genetic Analysis

DNA extraction was performed from peripheral blood using the Lahiri and Nurnberger method. For cell lysis, the sample was mixed with a special solution that includes 10 mM Tris-HCl pH 7.6, 10 mM KCl, 10 mM MgCl_2_ and 2 mM EDTA solution, and Nonidet P-40 (NP-40, Sigma-Aldrich, USA. Then, DNA was obtained by adding 0.8 mL of high-salt buffer containing 10 mM Tris-HCl pH 7.6, 10 mM KCl, 10 mM MgCl_2_, 0.4 M NaCl, 2 mM EDTA, and 50 μL of 10% SDS, and incubating for 10 min at 55 °C. After this, 0.30 mL of 6 M NaCl was added and the solution was centrifuged. Absolute ethanol followed by 70% ice-cold ethanol was then added to the supernatant to precipitate the DNA, then it was centrifuged at 4 °C and dried. Finally, DNA was resuspended in 0.5 mL of 10 mM Tris-HCl, 1 mM EDTA, pH 8.0 at 65 °C for 15 min, and stored at −80 °C in the biobank of our institution [28].

The *TRSP1* rs231150 *A/T*, *TRPS1* rs2737229 *A/C*, *TRIB1* rs2980880 *T/C*, and *TRIB1* rs2954029 *T/A* polymorphisms were analyzed using TaqMan assays using a QuantStudio 12K Flex Real-Time PCR system, under the following amplification program: 95 °C for 10 min, followed by 40 amplification cycles of 95 °C, for 15 s for denaturation, and 60 °C, for 1 min for annealing/extension, according to the manufacturers (Applied Biosystems, Foster City, CA, USA). Supplementary Table S1 displays information about these SNPs, including chromosome position, base change, minor allele, and gene location.

### 2.4. Statistical Analysis

The Hardy–Weinberg equilibrium was determined by chi-squared test. Data distribution was analyzed by the Shapiro–Francia test. Variables with normal distribution were compared by Student’s *t*-test and represented as mean ± SD. The Mann–Whitney U-test compared variables with non-normal distribution that were represented as median and interquartile interval [25th–75th]. Fisher’s exact test or chi-squared test was performed for categorical variables. The associations of the *TRSP1* rs231150 *A/T*, *TRPS1* rs2737229 *A/C*, *TRIB1* rs2980880 *T/C*, and *TRIB1* rs2954029 *T/A* gene polymorphisms with ACS were analyzed by logistic regression using models of inheritance, i.e., codominant, dominant, over-dominant (heterozygous), additive, and recessive [29,30]. Confounder variables including age, gender, body index mass (BMI), elevated blood pressure/hypertension, smoking habit, and diabetes incidence were included in the logistic regression analyses. The *p*-values were corrected (P) by the Bonferroni’s method. Results were presented as odds ratios (OR) and 95% confidence intervals. The statistical analyses were fixed to a power of 0.80 (OpenEpi available online, http://www.openepi.com/SampleSize/SSCC.htm (accessed on12 November 2023)). *p*-values < 0.05 were considered statistically significant.

Haplotypes and linkage disequilibrium (LD) were analyzed using Haploview version 4.1 [31]. This software, based on the international HapMap project database and the standard EM algorithm, estimates the haplotypes, considering the combination of alleles that can co-segregate due to their proximity in the chromosome [31].

### 2.5. Association of the TRSP1 and TRIB1 Genotypes with Cardiovascular Risk Factors

To investigate how the *TRSP1* (rs231150 *A/T*, rs2737229 *A/C*), and *TRIB1* (rs2980880 *T/C*, rs2954029 *T/A*) polymorphisms might affect triglycerides, total cholesterol, HDL-C, LDL-C, and glucose plasma levels, we only looked at the control group. We excluded the ACS patients from these analyses due to their use of anti-dyslipidemic drugs [32,33]. Individuals were grouped based on SNP genotypes, and comparisons were performed by ANOVA. For these analyses, non-normally distributed variables were logarithmically transformed. Variance homogeneity was evaluated by the Levine test and confirmed by the F test.

## 3. Results

### 3.1. Parameters of the Study Sample

Table 1 shows the demographic and biochemical characteristics of study groups. The proportion of males among ACS patients and the prevalence of cardiovascular risk factors were higher than those in the control subjects. In addition, total cholesterol, HDL-C, and LDL-C plasma levels were lower in the ACS patients than in the control group.

### 3.2. Association of TRSP1 and TRIB1 Polymorphisms with ACS

The genetic distribution of *TRSP1* and *TRIB1* SNPs in both groups were in Hardy–Weinberg equilibrium (*p* > 0.05). Except for the rs231150 SNP, the allele and genotype frequencies of the analyzed polymorphisms were different in patients when compared to controls (*p* < 0.05) (Supplementary Table S2). The analysis in Table 2 shows that carriers with one or two copies of the *A* allele of the *TRPS1* rs2737229 SNP have a higher probability of developing ACS based on different inheritance models. In addition, the *TRIB1* rs2980880 *T/C* polymorphism analysis showed that the carriers of the one or two copies of the *G* allele also had and increased risk of developing ACS, under the four inheritance models (Table 2). Finally, carriers of one or two copies of the *T* allele of the rs2954029 *T/A* SNP had an increased risk of developing ACS under co-dominant, recessive, and additive models (Table 2).

### 3.3. Haplotype Analysis

Table 3 presents the results of the haplotype analysis. This analysis was carried out as per the chromosome position; the SNPs of the *TRPS1* genes located in chromosome 8q23-3 region, rs231150 *A/T* and rs2737229 *A/C*, which were not in linkage disequilibrium (D’ = 0.17). The “*TA*” haplotype increased the risk of developing ACS (OR = 1.14, *p* = 0.035) (Table 3). Additionally, the study of the rs2980880 *T/C* and rs2954029 *T/A* polymorphisms of the *TRIB1* gene on chromosome 8q24-13 found two haplotypes (*TA* and *CT*) that showed significant differences between patients with ACS and controls (Table 3). The “*TA*” haplotype was more frequent in controls (39.9%) than in the ACS group (36.8%), (OR = 0.87, *p* = 0.030). Conversely, the “*CT*” haplotype was more frequent in patients (3.7%) than in controls (0.9%), indicating that this haplotype may represent a risk factor of ACS (OR = 4.24, *p* < 0.001). It is noteworthy that the rs2980880 *T/C* and rs2954029 *T/A* polymorphisms showed a moderated linkage disequilibrium (D’ = 0.75).

### 3.4. Association of TRSP1 and TRIB1 Polymorphisms with Plasma Lipids Concentrations

We explored the possible effect of the *TRSP1* (rs231150 *A/T* and rs2737229 *A/C*) and *TRIB1* (rs2980880 *T/C* and rs2954029 *T/A*) polymorphisms on plasma lipid profile in control subjects. It is important to emphasize that this group did not receive any drug affecting the lipid profile levels (n = 1051), whereas ACS patients received anti-dyslipidemic drugs [32,33]. In this context, despite the lack of association of rs231150 *A/T* SNP with ACS incidence, the *TA* genotype was associated with high LDL-C concentrations (*p* < 0.05). In addition, homozygous carriers of the rs231150 *AA* and rs2737229 *AA* genotypes were related to low triglyceride levels (*p* < 0.05) (Table 4). Also, the homozygous carriers of the rs231150 *A* allele, as well as the rs2954029 *T* allele, had increased HDL-C plasma concentrations (*p* < 0.05) (Table 4). Additionally, the rs2980880 *CC* genotype was linked to higher total cholesterol and LDL-C plasma levels (*p* < 0.05) (Table 4).

## 4. Discussion

In this work, we investigated whether the *TRSP1* (rs231150 *A/T* and rs2737229 *A/C*) and *TRIB1* (rs2980880 *T/C* and rs2954029 *T/A*) SNPs are associated with incidence of ACS and with plasma lipid profile. These SNPs are located in the *TRSP1* and *TRIB1* genes that encode the transcriptional repressor GATA binding 1 and tribbles pseudokinase 1 proteins, respectively, which are associated with the predisposition to coronary heart disease, and with plasma lipid profile levels in different populations [4,5,6,7,8,9,10]. We determined that minor alleles (rs2737229 *A*, and rs2980880 *C*, and rs2954029 *T*) conferred a risk of developing ACS. The association of these SNPs with the prevalence of ACS in other populations is scarce. In addition, the association of these SNPs with CHD is controversial in different populations; in our study, the “*A*” allele of the *TRSP1* rs2737229 *A/C* SNP was associated with an increase in ACS risk. Previous studies have related this polymorphism with plasma lipid levels but not with a CHD risk [4,10,34]. Moreover, the sub-analysis of the controls grouped by genotype showed that the rs2737229 *AA* homozygous carriers had a significantly lower level of plasma triglycerides than *TT* homozygotes, which may be considered beneficial in terms of lipidic risk factors of CHD. Experimental studies demonstrated that *TRSP1* decreased the expression of the scavenger receptor B1 (SR-BI) [13,14], which plays an important role in the recognition of lipoproteins, including intermediate-density lipoproteins (IDL) [35]. Therefore, we speculate that this polymorphic site is an ethnic trait that interacts with other genes or environmental factors [10] to confer the increased risk of CHD, even if it likely has a potential effect on decreasing triglyceride plasma levels. This hypothesis merits exploration in other populations.

Focusing on the *TRIB1* rs2954029 *A/T* SNP, having two copies of the *T* allele was linked to acute coronary syndrome (ACS) and, surprisingly, to higher levels of HDL-C. Recent studies showed that the allele *T* was associated with the prevalence of dyslipidemia [4,17,36,37,38] and ischemic heart disease or myocardial infarction [4,17,36,37,38]; also, the association of the T allele with higher levels of HDL-C is consistent in populations with different ethnicities [37,38]. On the other hand, earlier studies showed that the *A* allele influences total cholesterol, triglycerides, and LDL-C levels and is linked to a higher risk of developing coronary heart disease (CHD) and ischemic stroke [10,34,39]. Such paradoxical results stress the proposed functional effect of this polymorphism on hepatic lipid synthesis [40]. Specific functional studies are needed to elucidate whether this polymorphism affects the protein gene expression. For the *TRIB1* rs2980880 *T/C* polymorphism, the *C* allele was linked to a higher risk of ACS and higher levels of total cholesterol and LDL-C in our population. In line with our finding, Zhang et al. determined that the rs2980880 *C* allele is associated with high HDL-C levels and with an increased risk of developing ischemic stroke, but not with CHD in the Chinese population [10]. Finally, haplotype analyses showed that the “*TA*” haplotype conformed by the *TRSP1* (rs231150 *A/T* and rs2737229 *A/C*) and the “*CT*” haplotype conformed by the *TRIB1* (rs2980880 *T/C* and rs2954029 *T/A*) SNPs were associated with risk of developing ACS; as far we know, there are no studies that showed similar haplotypes associations with ACS. These findings support the potential usefulness of the rs2737229 *A*, rs2980880 *C*, and rs2954029 *T* alleles as genetic marker of ACS risk.

On the other hand, the genotype-based subanalyses performed in the control group showed that *TRSP1* (rs231150 *A/T* and rs2737229 *A/C*) and *TRIB1* (rs2980880 *T/C* and rs2954029 *T/A*) SNPs are associated with the plasma lipid levels; the rs231150 *AA* and rs2954029 *TT* genotypes were associated with higher levels of HDL-cholesterol. Importantly, the effect of these genotypes on HDL-C plasma levels in our study may be exacerbated by the fact that Mexicans are prone to hypoalphalipoproteinemia [41]. In addition, rs231150 *AA* and rs2737229 *AA* genotypes favor lower triglyceride concentrations than rs231150 *TT* and rs2737229 *CC* genotypes. In this context, experimental studies have shown that the overexpression of Trib1 in mice reduced the hepatic triglycerides, and knockout of *TRIB1* exhibits significant increases in plasma triglycerides [40]. In humans, *TRIB1* regulates the *MLXIPL* gene, which encodes to the carbohydrate-response element-binding protein (ChREBP), which has an important role in hepatic lipogenesis [17]. Therefore, the rs231150 *AA* and rs2737229 *AA* genotypes likely enhance Trib1 expression or activity. We need further studies to validate this hypothesis.

Finally, the effect of transcriptional repressor GATA binding 1 (*TRSP1*) and tribbles pseudokinase 1 (*TRIB1*) on plasma lipid concentrations has not yet been fully determined. We observed that the contribution of the studied SNPs in ACS patients and plasma lipid concentrations remains controversial, probably because of the ethnic origin. Particularly, the rs231150 *A*, rs2737229 *A*, rs2980880 *C,* and rs2954029 *A* minor allele frequencies were low in Mexican mestizos compared to the Caucasian population (Supplementary Table S3). Additionally, the frequencies of the rs2737229 *A* and rs2954029 *A* alleles were low in Mexican mestizos compared to the Asian population. Also, the distribution of the rs2980880 *C* allele was higher than in ours, but the rs231150 *A*, rs2737229 *A*, and rs2954029 *A* alleles were very low in the African population compared to other ethnic groups (Supplementary Table S3). As observed, the distribution of *TRSP1* and *TRIB1* polymorphisms is different compared with other populations, so we suggest that the impact of these SNPs on ACS and other heart diseases should be studied in larger research projects that include patients from various backgrounds.

### Limitations of the Study

We recognize that the proportion of men to women in the ACS patients was higher than in the control group. Although this limitation was viewed as a confusing variable, it should still be taken into account when interpreting the study findings. Also, our results did not establish a cause–effect relationship between *TRSP1* and *TRIB1* polymorphisms and ACS or with dyslipidemia; the study only showed a statistical correlation between minor allele risk of developing ACS and plasma lipids levels. Therefore, we need more genetic studies in various ethnic groups and experiments on mRNA expression to confirm that *TRSP1* and *TRIB1* polymorphisms are useful in clinical practice.

## 5. Conclusions

The *TRSP1* rs2737229, *TRIB1* rs2980880, and *TRIB1* rs2954029 SNPs were associated with an increased risk of ACS. Furthermore, the same SNPs were associated with dyslipidemia in Mexican individuals. Finally, according to our findings and the polymorphisms distribution in our population, these SNPs should be researched in other ethnic groups to see if they are a risk factor for ACS and other heart diseases.

## Figures and Tables

**Table 1:** Parameters of the individuals studied.

Characteristics		ACS Patients (n (%)) (n = 1262)	Controls (n (%)) (n = 1051)	*p*-Value *
Age (years)		59.1 ± 10.8	51.3 ± 8.9	<0.001
BMI (kg/m^2^)		27.6 ± 4.21	28.2 ± 4.05	<0.001
Sex, n (%)	Male	1016 (80.5)	426 (40.5)	<0.001
	Female	246 (19.4)	625 (59.4)	<0.001
Elevated blood pressure, n (%)	Yes	719 (57)	193 (18)	<0.001
Type 2 diabetes mellitus, n (%)	Yes	737 (58)	62 (6)	<0.001
Smoking, n (%)	Yes	612 (48)	231 (22)	<0.001
Blood pressure (mmHg)	Systolic	130 [115–150]	112 [103–122]	<0.001
	Diastolic	80 [70–90]	70 [65–76]	<0.001
Glucose (mg/dL)		136 [109–200]	90 [84–97]	<0.001
Total cholesterol (mg/dL)		157 [126–190]	190 [167–211]	<0.001
HDL-C (mg/dL)		37 [31–44]	45 [36–55]	<0.001
LDL-C (mg/dL)		97 [71–126]	116 [95–134]	<0.001
Triglycerides (mg/dL)		142 [107–193]	146 [108–203]	0.275

Results are the mean ± SD or median and interquartile interval [25th–75th]. * Mann–Whitney U or Student’s *t* test (continuous variables) and χ^2^ (categorical values). ACS: acute coronary syndrome; BMI: body mass index.

**Table 2:** Association of the *TRIB1* and *TRPS1* polymorphisms with ACS accordance to the inheritance models.

SNP (rsID-Number)/ * Inheritance Model	Genotype	ACS Patients n = 1262 (n(%))	Controls n = 1051 (n(%))	OR (95%CI)	pC
*TRPS1* rs2737229 *A/C*					
Co-dominant	*CC* *AC* *AA*	420 (33.3) 590 (46.8) 252 (20.0)	371 (35.3) 513 (48.8) 167 (15.9)	1.41 (1.06–1.87)	0.046
Dominant	*CC* *AC + AA*	420 (33.3) 842 (66.7)	371 (35.3) 680 (64.7)	1.15 (1.07–1.41)	0.171
Recessive	*CC + AC* *AA*	1010 (80.0) 252 (20.0)	884 (84.1) 167 (15.9)	1.36 (1.06–1.74)	0.017
Over-dominant	*CC + AA* *AC*	672 (53.2) 590 (46.8)	538 (51.2) 513 (48.8)	0.95 (0.79–1.15)	0.598
Additive	--------	---------	-----------	1.17 (1.02–1.34)	0.026
*TRIB1* rs2980880 *C/T*					
Co-dominant	*TT* *TC* *CC*	668 (52.9) 481 (38.1) 113 (8.9)	590 (56.1) 393 (37.4) 68 (6.5)	1.84 (1.16–2.90)	0.015
Dominant	*TT* *TC + CC*	668 (52.9) 594 (47.1)	590 (56.1) 461 (43.9)	1.34 (1.06–1.70)	0.015
Recessive	*TT + TC* *CC*	1149 (91.0) 113 (8.9)	938 (93.5) 68 (6.5)	1.66 (1.07–2.59)	0.024
Over-dominant	*TT + CC* *TC*	781 (61.9) 481 (38.1)	658 (62.6) 393 (37.4)	1.17 (0.92–1.49)	0.209
Additive	--------	---------	-----------	1.31 (1.09–1.58)	0.004
*TRIB1* rs2954029 *T/A*					
Co-dominant	*AA* *AT* *TT*	485 (38.4) 572 (45.3) 205 (16.2)	432 (41.1) 485 (46.1) 134 (12.8)	1.42 (1.08–1.87)	0.037
Dominant	*AA* *AT + TT*	485 (38.4) 777 (61.6)	432 (41.1) 619 (58.9)	1.14 (0.95–1.36)	0.161
Recessive	*AA + AT* *TT*	1057 (83.8) 205 (16.2)	917 (87.2) 134 (12.8)	1.38 (1.07–1.78)	0.012
Over-dominant	*AA + TT* *AT*	690 (54.7) 572 (45.3)	566 (53.9) 485 (46.1)	0.97 (0.81–1.15)	0.699
Additive	--------	---------	-----------	1.16 (1.02–1.32)	0.024

* Inheritance models based on the minor allele; the co-dominant compares the homozygous minor allele carriers to major allele homozygotes. Dominant model compares the homozygous major allele carriers to the heterozygotes and minor allele homozygotes. Recessive model groups heterozygotes and major allele homozygotes and compares them to minor allele homozygotes. Over-dominant model compares major and minor allele homozygotes vs. heterozygotes. Additive model compares the major allele carriers to the subgroup of heterozygotes and minor allele homozygotes. Logistic regression included age, gender, BMI, smoking, elevated blood pressure/hypertension, and diabetes incidence. SNP: Single nucleotide polymorphism; ACS: Acute coronary syndrome; OR: odds ratio; CI: confidence interval; pC: corrected *p*-value.

**Table 3:** Haplotypes distribution of *TRPS1* and *TRIB1* polymorphisms in the study groups.

* Polymorphic Site	ACS Patients n = 1262	Controls n = 1051	OR	95%CI	*p*
Block Haplotype	Hf (%)	Hf (%)			
*T* *C*	0.377	0.390	0.94	0.84–1.06	0.385
*A* *A*	0.219	0.210	1.05	0.91–1.21	0.462
*T* *A*	0.215	0.193	1.14	1.00–1.31	0.035
*A* *C*	0.189	0.207	0.89	0.77–1.03	0.062
Block Haplotype	Hf (%)	Hf (%)			*p*
*T* *A*	0.368	0.399	0.87	0.77–0.98	0.030
*T* *T*	0.352	0.349	1.01	0.89–1.14	0.851
*C* *A*	0.243	0.243	1.00	0.87–1.14	0.984
*C* *T*	0.037	0.009	4.24	2.62–7.13	<0.001

Analysis of haplotypes was performed with Haploview software, version 4.1 [31]. * The polymorphisms order and the allele combination of the haplotypes is according to the position in the chromosomes 8q23.3 (rs231150 *A/T*–rs2737229 *A/C*) and 8q24.13 (rs2980880 *C/T*–rs2954029 *T/A.* Hf: Haplotype frequency; ACS: acute coronary syndrome.

**Table 4:** Plasma lipids concentration according to the genotypes of the rs231150 *A/T*, rs82737229 *A/C,* rs12980880 *G/A,* and rs2954029 *T/A* polymorphisms in the control group.

*TRPS1*	rs231150 *A/T*			
	*TT* (n = 345)	*AT* (n = 535)	*AA* (n = 171)	*p*-value *
Parameters				
Glucose (mg/dL)	89 [83–96]	91 [85–98]	88 [83–96]	0.263
Total cholesterol (mg/dL)	187 [164–208]	192 [169–215]	189 [169–210]	0.070
HDL-C (mg/dL)	44 [36–53]	45 [36–55]	46 [38–60]	0.036
LDL-C (mg/dL)	113 [93–133]	118 [99–137]	114 [94–131]	0.035
Triglycerides (mg/dL)	148 [108–205]	149 [113–202]	128 [97–191]	0.041
*TRPS1*	rs2737229 *A/C*			
	*CC* (n = 371)	*AC* (n = 513)	*AA* (n = 167)	p-value
Parameters				
Glucose (mg/dL)	90 [84–98]	90 [85–97]	88 [81–94]	0.311
Total cholesterol (mg/dL)	189 [167–213]	191 [169–211]	188 [164–209]	0.169
HDL-C (mg/dL)	45 [36–54]	45 [36–55]	45 [37–57]	0.333
LDL-C (mg/dL)	113 [95–135]	118 [96–134]	113 [93–133]	0.232
Triglycerides (mg/dL)	156 [115–210]	147 [107–204]	125 [99–181]	<0.001
*TRIB1*	rs2980880 *C/T*			
	*TT* (n = 590)	*TC* (n = 393)	*CC* (n = 68)	*p*-value
Parameters				
Glucose (mg/dL)	90 [84–96]	90 [84–98]	90 [84–95]	0.751
Total cholesterol (mg/dL)	188 [164–209]	191 [170–217]	197 [170–216]	0.037
HDL-C (mg/dL)	45 [36–56]	45 [36–54]	43 [36–54]	0.573
LDL-C (mg/dL)	114 [94–132]	116 [98–137]	122 [99–142]	0.025
Triglycerides (mg/dL)	144 [107–192]	147 [110–214]	152 [101–190]	0.134
*TRIB1*	rs2954029 *T/A*			
	*AA* (n = 432)	*AT* (n = 485)	*TT* (n = 134)	p-value
Parameters				
Glucose (mg/dL)	91 [84–97]	89 [83–96]	90 [85–99]	0.150
Total cholesterol (mg/dL)	190 [167–212]	190 [169–210]	190 [163–211]	0.848
HDL-C (mg/dL)	45 [36–54]	44 [36–54]	48 [38–58]	0.017
LDL-C (mg/dL)	116 [97–133]	116 [95–135]	114 [95–133]	0.732
Triglycerides (mg/dL)	145 [109–204]	148 [107–205]	131 [101–189]	0.223

Results are the mean ± SD or median and interquartile interval [25th–75th]. Non-normally distributed variables were logarithmically transformed for * ANOVA analysis. Abbreviations: HDL: high-density lipoprotein, LDL: low-density lipoprotein.

## Data Availability

The data shown in this work are available on request from the corresponding author.

## Supplementary Materials

The following supporting information can be downloaded at: https://www.mdpi.com/article/10.3390/biology14060606/s1, Table S1: Information of the studied polymorphism tested, Table S2: Allele and genotype frequencies of *TRPS 1* and *TRIB 1* genes polymorphisms in ACS patients and healthy controls, and Table S3: Allele (af) frequencies of the *TRSP1* and *TRIB1* polymorphisms in different populations..
