# Supplementary material for: TRIB1 and TRPS1 Gene Polymorphisms Are Associated with the Incidence of Acute Coronary Syndrome and Plasma Lipid Concentrations
Source: Biology (Basel). 2025 May 26;14(6):606. doi: 10.3390/biology14060606 (PMC12189847; doi:10.3390/biology14060606)
Supplement: Supplementary file 1 [file biology-14-00606-s001.zip › biology-3598596-supplementary.pdf]

Supplementary Table S1. Information of the studied polymorphism tested

Gene symbol	SNP (rsID-number)[a,b]	Chromosome	Chromosome position	Alleles major/minor	Location in gene
TRPS1	rs231150	8q23.3	115408099	*T/A*	Downstream Transcript Variant
TRPS1	rs2737229	8q23.3	115636338	*C/A*	Intron Variant
TRIB1	rs2980880	8q24.13	125468730	*T/C*	Intron Variant
TRIB1	rs2954029	8q24.13	125478730	*A/T*	Intron Variant

[a] Single Nucleotide Polymorphisms (rsID-number) in database dbSNP; [b] Given name according to NCBI, Current Build 156 (released September 21, 2022) https://www-ncbi-nlm-nih-gov.pbidi.unam.mx:2443/snp/?term=rs231150.

Supplementary Table S2. Allele and genotype frequencies of *TRPS 1* and *TRIB 1* genes polymorphisms in ACS patients and healthy controls

SNP (rsID-number)	ACS n=1262, n (%)	Controls n=1051. n (%)	*p
rs231150 *A/T*			
Allele			
T	1494 (59.1)	1225 (58.2)	
A	1030 (40.8)	877 (41.7)	NS
Genotype			
TT	442 (35.0)	345 (32.8)	
AT	610 (48.3)	535 (50.9)	NS
AA	210 (16.6)	171 (16.3)	
rs2737229 *A/C*			
Allele			
C	1430 (56.6)	1255 (59.7)	
A	1094 (43.3)	847 (40.2)	0.036
Genotype			
CC	420 (33.3)	371 (35.3)	
AC	590 (46.8)	513 (48.8)	
AA	252 (20.0)	167 (15.9)	0.011
rs2980880 *C/T*			
Allele			
T	1817 (71.9)	1573 (74.8)	
C	707 (28.0)	529 (25.1)	0.029
Genotype			
TT	668 (52.9)	590 (56.1)	
TC	481 (38.1)	393 (37.4)	
CC	113 (8.9)	68 (6.5)	0.026
rs2954029 *T/A*			
Allele			
A	1542 (61.0)	1349 (64.1)	
T	982 (38.9)	753 (35.8)	0.031
Genotype			
AA	485 (38.4)	432 (41.1)	
AT	572 (45.3)	485 (46.1)	
TT	205 (16.2)	134 (12.7)	0.017

SNP, Single nucleotide polymorphism, ACS, Acute coronary syndrome. Data are shown as n and frequency expressed as percentage. *chi-square test. NS: No significant

Supplementary Table S3. Allele (af) frequencies of the *TRSP1* and *TRIB1* polymorphisms in different populations.

SNP/Alleles	Mexican		Caucasian*		Asian*		African*		References
TRSP1 rs231150 *A/T*									
	(n=1051)		(n=502)		(n=504)		(n=661)		
Allele	n	af	n	af	n	af	n	af	
T	1225	58.2	475	47.2	575	57.0	935	70.7	Present study[A]
A	877	41.7[A]	531	52.8	433	43.0	387	29.3	NCBI*
TRSP1 rs2737229 *A/C*									
	(n=1051)		(n=503)		(n=504)		(n=661)		
Allele	n	af	n	af	n	af	n	af	
C	1255	59.7	270	26.8	354	35.1	1205	91.1	Present study[B]
A	847	40.2 [B]	736	73.2	654	64.9	117	8.9	NCBI*
TRIB1 rs2980880 *C/T*									
	(n=1051)		(n=503)		(n=504)		(n=661)		
Allele	n	af	n	af	n	af	n	af	
T	1573	74.8	671	66.7	734	72.8	872	66.0	Present study[F]
C	529	25.1 [C]	335	33.3	274	27.2	450	34.0	NCBI*
TRIB1 rs2954029 *T/A*									
	(n=1051)		(n=503)		(n=504)		(n=661)		
Allele	n	af	n	af	n	af	n	af	
A	1349	64.1	555	55.2	452	44.8	938	71.0	Present study[G]
T	753	35.8 [D]	451	44.8	556	55.2	384	29.0	NCBI*

SNP, Single nucleotide polymorphism; *NCBI, National Center for Biotechnology Information (https://www.ensembl.org/index.html (accessed on 20 January 2025)).

[A] The distribution of the rs231150 *A*, was low in Mexican mestizos, Asian, and African compared to Caucasian population.

[B] The distribution of the rs2737229 *A* allele in African and Mexican mestizos, was low compared to Asian and Caucasian populations.

[C] The distribution of the of the rs2980880 *C* allele in Mexican mestizos and Asian populations was low compared to African and Caucasian populations.

[D] The distribution of the rs2954029 *A* allele in Mexican Mestizos and African was low when compared to Asian and Caucasian populations.

[A,D] The distribution of the rs231150 *A* and rs2980880 *C* alleles in Mexican mestizos and Asian, was similar compared to Caucasian population.
